# Confirmatory factor analysis of the Appraisal of Self-Care Agency Scale - Revised^1^

**DOI:** 10.1590/1518-8345.1378.2856

**Published:** 2017-01-30

**Authors:** Thaís Santos Guerra Stacciarini, Ana Emilia Pace

**Affiliations:** 2 PhD, RN, Hospital de Clínicas, Universidade Federal do Triângulo Mineiro, Uberaba, MG, Brazil.; 3 PhD, Associate Professor, Escola de Enfermagem de Ribeirão Preto, Universidade de São Paulo, WHO Collaborating Centre for Nursing Research Development, Ribeirão Preto, SP, Brazil.

**Keywords:** Validation Studies, Psychometrics, Factor Analysis, Diabetes Mellitus, Self-Care

## Abstract

**Objective:**

to analyze the factor structure of the Appraisal of Self-Care Agency Scale-Revised (ASAS-R), adapted for Brazil.

**Method:**

methodological study conducted with 150 individuals with diabetes mellitus cared for by the Family Health Strategy, most of whom are elderly with low educational levels. The test of the hypothesis concerning the confirmatory factor composition of the ASAS-R was performed using latent variables structural equations.

**Results:**

the model’s goodness-of-fit indexes were satisfactory (χ^2^ = 259.19; χ^2^/g.l = 2.97, p < 0.001; GFI = 0.85; RMR = 0.07; RMSEA = 0.09); the factor loads were greater than 0.40; and most item-to-factor-correlations presented moderate to strong magnitude (0.34 to 0.58); total alpha value was 0.74, while the alpha of the three factors were 0.69, 0.38 and 0.69, respectively.

**Conclusion:**

the scale’s factor structure presented satisfactory validity and reliability results, with the exception of one factor. Application of this scale to samples of the general population is desirable in order to strengthen analyses of internal consistency and the dimensionality of the factor structure. This study is expected to contribute to further studies addressing the self-care agency construct and the development of the ASAS-R.

## Introduction

Self-care agency, a central concept in Orem’s Self-Care Deficit Theory (SCDT), is defined as one’s ability to exercise self-care in order to maintain life, health and wellbeing. It is a complex ability acquired over the course of life, based on repeated behavior on a daily basis and is influenced by cultural aspects and background, personal skills and limitations, life experiences, health status, and resources available^1^.

The development of self-care agency enables an individual to discern between factors that need to be controlled and taken care of, decide what one can do and what one needs to do, recognize one’s own needs, assess personal and environmental resources, and undertake actions that meet one’s self-care needs^1^.

According to the SCDT, the relationship between one’s self-care ability and needs is essential to establishing the actions one should perform and those actions one has the ability to develop in order to maintain health and prevent and manage diseases^1^.

This understanding is important to assessing the self-care ability of people with chronic diseases, especially *diabetes mellitus*, which is a condition that requires great responsibility and commitment, especially from those taking insulin^2^ ^-^ ^4^, to carry on with the therapeutic regimen that includes behavioral modification of daily activities^5^. From this perspective, assessing an individual’s personal ability to perform self-care has been widely studied to highlight the individual’s performance in preventing and managing *diabetes mellitus* ^4^ ^,^ ^6^ ^-^ ^8^.

The Appraisal of Self-Care Agency Scale - Revised was adapted and validated in Brazil with a sample of individuals with DM taking insulin, though it is not a specific scale^9^.

The conceptual basis for the development of this scale was the Self-Care Deficit Theory developed by Orem^1^. The scale’s items concerning the concept of self-care agency were based on empowering traits or power components (specific personal abilities to perform self-care) and operational traits (ability to organize personal and environmental resources that might be significant in self-care)^1^ ^,^ ^10^.

The revised version was chosen by Sousa^11^ to be adapted to Portuguese with a sample of Brazilian individuals because it presents a better fit index, greater reliability (total Cronbach’s alpha = 0.89) and better validation results in comparison to its original version^9^. The changes performed in the revised version included the exclusion of nine items and the presentation of three factors that were not reported in the original version^11^.

The translation and adaptation process of the ASAS-R in Brazil followed the stages recommended in the literature^12^. After the translation and adaptation process, the scale was submitted to analysis of the distribution of frequencies of items’ responses, reliability (internal consistency and product-moment correlation), reproducibility (test-retest and inter-observer), and construct validity (convergent and discriminant)^9^.

The results of the psychometric analysis show that the one-dimension structure of the adapted scale is reliable (total Cronbach’s alpha = 0.74), reproducible (test-retest p < 0.001 and inter-observer p < 0.001) and valid (confirmed the correlation hypotheses with the constructs depression and perceived health status and between distinct groups)(9). The hypotheses were based on Orem’s theoretical framework in regard to factors that affect the development and maintenance of self-care agency(1).

Therefore, aiming to continue the psychometric testing of the ASAS-R with a sample of Brazilian individuals with *diabetes mellitus*, we performed a confirmatory factor analysis of the scale to verify whether its dimensions present reliable and valid representations.

## Method

This methodological study with quantitative approach was conducted with a sample of 150 Brazilian individuals with type 2 *diabetes mellitus* taking insulin and cared for by three Family Health Strategy units in a city in the interior of Minas Gerais, Brazil, an important economic hub in the region and a reference center in health and education.

Data were collected from September 2011 to February 2012. Inclusion criteria were: both sexes, being 18 years old or older; having been diagnosed with type 2 *diabetes mellitus*, enrolled in the FHS, and taking insulin for more than one year; and being able to answer the instruments’ questions. Data were collected through an interview held at the participants’ home or during consultations.

The ASAS-R contains 15 items assessed on a five-point Likert scale, of which only one alternative may be chosen. Scores range from 1 to 5, where: 1 - “totally disagree”; 2 - “disagree”; 3 - “I do not know”; 4 - “agree”; and 5 - “totally agree”. Four out of the 15 questions refer to negative aspects (ASAS-R 4, 11, 14 and 15)^9^ ^,^ ^11^.

The total score ranges from 15 to 75; the higher the score, the greater one’s operational self-care ability^9^. The three factors were denoted: “Having power for self-care”, composed of items 1, 2, 3, 5, 6 and 10; “Developing power for self-care”, items 7, 8, 9, 12 and 13; and “Lacking power for self-care”, items 4, 11, 14 and 15^1^ ^,^ ^9^.

Data were coded, categorized and typed into an Excel spreadsheet, then exported and processed using the Statistical Package for the Social Sciences version 16.0 to obtain descriptive analyses, variability (standard deviation (SD), minimum and maximum), measures of central tendency (mean, median) and psychometric analyses (reliability and factor validation).

Cronbach’s alpha was used for the reliability analysis; acceptable values for a scale with a small number of items were between 0.50 and 0.90^13^. Pearson’s product-moment correlation less than 0.30 was considered weak with poor clinical applicability; between 0.30 and 0.50 was considered moderate; and greater than 0.50 was considered strong^14^. The significance level was established at 0.05.

In the confirmatory factor analysis, overall fit of the hypothesized factorial model and estimation of the construct’s effects on measured variables were considered. Hypothesis testing for the factorial composition of the ASAS-R scale was implemented using latent variable structural equations. 

The following indexes were analyzed to verify the model’s goodness of fit^15^: Chi-square test (χ^2^), with significance greater than 0.05; Chi-square ratio (χ^2^/g.l), with acceptable values below 2.0; Goodness of Fit Index (GFI), with acceptable values equal to or greater than 0.85; GFI Adjusted for Degrees of Freedom (AGFI), with acceptable values equal to or greater than 0.80; Root Mean Square Residual (RMR), with acceptable values equal to or greater than 0.10; Root Mean Square Error of Approximation (RMSEA), with acceptable values equal to or lower than 0.08; Bentler’s Comparative Fit Index (CFI), with acceptable values equal to or greater than 0.90; and Bentler & Bonett’s Non-normed Fit Index (NNFI), with acceptable values equal to or greater than 0.90.

At least three adequacy indexes with values greater than their references were considered in analyzing the goodness of fit of data to the proposed factors^16^. The estimation method used was maximum likelihood with a minimum of ten observations per item, which presented univariate normality of items^17^.

Additional tests for the analysis of the adapted scale (Wald and Lagrange multiplier tests and exploratory analysis) were used to identify a factor structure with more robust results, if there were a weak item-to-factor correlation, low internal consistency of factors or overall fit of the hypothesized factor model with unsatisfactory or modest values compared to the original version. 

Wald’s test verifies the extent to which the removal of an item influences the model’s Chi-square statistics. Items can be removed without affecting future results when change is not significant^15^. The Lagrange’s multiplier test verifies the need to reallocate an item to another factor to improve correlation among the items within the same factor. Similar to the Wald’s test, it shows how much an item reallocated to a new factor will influence the Chi-square statistics^15^.

In the exploratory factor analysis, the Kaiser-Meyer-Olkin (KMO) index and Bartlett’s sphericity test (BTS) are used to assess how adequate the sample size and the factor analysis are to test the null hypothesis of the identity matrix, that is, to verify that there is no cross-correlation among variables and that all off-diagonal correlations are zero. The values expected for the KMO test are between 0.5 and 1 and p < 0.5 for the BTS^17^.

In the analysis of the principal components, the factors that obtained eigenvalues (total variance explained for each factor) greater than one were selected and interpreted in a scree plot. The extraction of principal factors is performed after Varimax orthogonal rotation and Kaiser’s criterion^17^.

The programs used for the confirmatory and exploratory analyses were the Statistical Analysis System (SAS) for Windows, version 8.02 and the Statistical Package for the Social Sciences (SPSS) version 16.0, respectively.

The study was approved by the city’s Family Health Strategy coordination and the Institutional Review Board at the Federal University of Triângulo Mineiro (Protocol No. 1602/2010). The participants signed free and informed consent forms authorizing the collection and use of data.

Authorization to adapt the ASAS-R for Brazil was provided by the author Dr. Valmi D Sousa, in 2009, who signed an agreement form.

## Results

A total of 150 people took part in the analysis of the psychometric properties of ASAS-R. Their sociodemographic and clinical characteristics are presented in Table 1.

**Table 1:** Distribution of individuals with *diabetes mellitus* 2 taking insulin and with care provided by the Family Health Strategy according to sociodemographic and clinical characteristics (n = 150). Uberaba, MG, Brazil, 2012

Sociodemographic and clinical characteristics	n	%	Interval	Median	Mean	SD
Sex						
Female	83	55.3				
Male	67	44.7				
Age group (years)			18 - 94	64	58.6	16.4
< 60	56	37.3				
≥ 60	94	62.7				
Marital status						
Married/stable union	72	48.0				
Single	39	26.0				
Widowed	33	22.0				
Divorced/separated	6	4.0				
Occupation						
Retired/pensioner	76	50.7				
Employed	48	32.0				
Homemaker	20	13.3				
Unemployed	1	0.7				
Student	5	3.3				
Education						
Illiterate	17	11.3				
No education/can write and read	14	9.3				
1-9 years of school	74	49.4				
≥ 9 years of school	45	30.0				
Income (minimum wage*) Family income			0- 2,200.00	1,000.00	924.63	556.75
Time since DM diagnosis (years)			1 - 41	13	10.5	8.78
Time taking insulin (years)			1 - 40	5	6.41	6.24

* Minimum wage at the time was R$ 545.00

Table 1 shows that elderly individuals, retired, married with a low educational level and low income, predominate. Table 2 presents the results concerning the item to factor coefficient of correlation and reliability of factors.

**Table 2:** Presentation of results concerning the confirmatory factor analysis of the adapted version Appraisal of Self-Care Agency Scale-Revised. Uberaba, MG, Brazil, 2012

Factor/Item	Item-factor correlation	Cronbach’s α of excluded item
Factor 1: having power for self-care		
α_total_ = 0.69		
Item 1	0.41	0.61
Item 2	0.40	0.62
Item 3	0.58	0.55
Item 5	0.48	0.58
Item 6	0.25	0.72
Item 10	0.34	0.65
Factor 2. Developing power for self-care		
α_total_ = 0.38		
Item 7	0.44	0.07
Item 8	0.25	0.30
Item 9	0.17	0.36
Item 12	0.39	0.20
Item 13	-0.18	0.56
Factor 3. Lacking power for self-care		
α_total_ = 0.69		
Item 4	0.40	0.70
Item 11	0.43	0.60
Item 14	0.48	0.57
Item 15	0.51	0.51

Analysis of item reliability, as described in Table 2, revealed satisfactory internal consistency for factors 1 and 3 (alpha = 0.69). Most correlations among the items of each of the three factors were from moderate to strong magnitude (r = 0.34 to r = 0.58), except for items 6, 8, 9 and 13. Among these four items with weak correlation (r < 0.30), three (8, 9 and 13) are contained in factor 2 “Developing power” (Table 2).

Analysis concerning the correlation among factors revealed correlations of weak magnitude between the factors “Having power” and “Lacking power” (r = 0.21) and moderate magnitude between the factors “Having power” and “Developing power” and between “Developing power” and “Lacking power” (r = 0.44). On the other hand, the correlations of each of the three factors (Having, Developing and Lacking power) with the totality of items presented results of strong magnitude (0.71; 0.80 and 0.76), respectively. 

**Figure 1:**
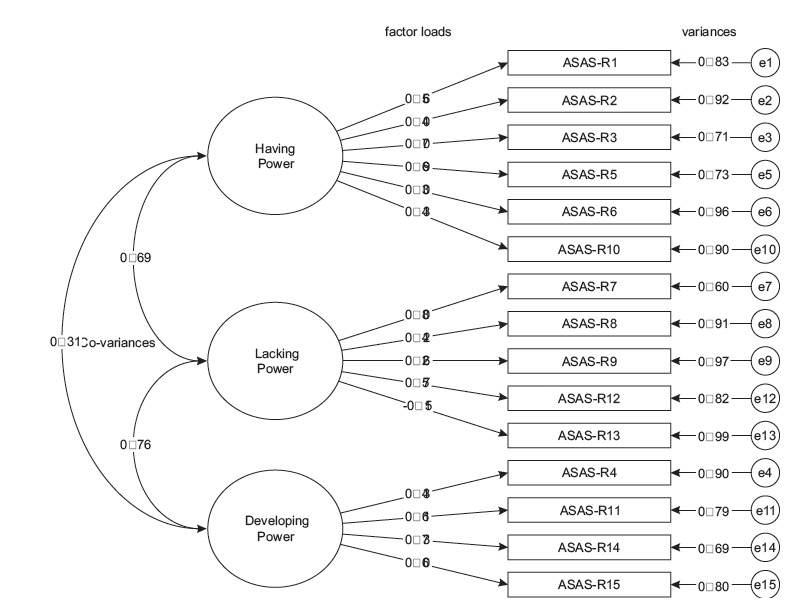
Path diagram of the confirmatory analysis results concerning the adapted version of the Appraisal of Self-Care Agency Scale-Revised.

The graphical expression of the path diagram, Figure 1, shows the factor loads of the observed variables (ASAS-R 1 to ASAS-R 15) in the latent variables (Having, Developing and Lacking power for self-care), as well as the co-variances between factors and items variances. In general, the results of the factor loads presented good values, that is, greater than 0.40, in their factor. The symbol represented by letter e, called error, is not represented by numerical values.

The overall fitting results were χ^2^ = 259.19; χ^2^/g.l = 2.97, p < 0.001; GFI = 0.85; AGFI = 0.77; RMR = 0.07; RMSEA = 0.09; CFI = 0.68 and NNFI = 0.61. These results show the model’s satisfactory fitting based on adequacy criteria GFI, RMR and RMSEA. AGFI was close to its reference value 0.80.

The Wald test showed that the exclusion of item 13, “I seek help when I am unable to take care of myself,” reduced the model’s Chi-square (χ^2^/g.l = 2.714) but did not affect future results, as it was not significant (p = 0.099). The Lagrange multiplier test suggested the reallocation of item ASAS-R 8 in factor 1 and item ASAS-R 10 in factor 2.

A new confirmatory factor analysis with the changes that resulted from the Wald and Lagrange tests showed an increase in the factor loads of the items in the factors, though not significant. Small changes were observed in the χ^2^ statistics (χ^2^ =200.33; χ^2^/g.l = 2.707; p < 0.001) and measures of goodness of fit (GFI = 0.86; AGFI = 0.80; RMR = 0.07; RMSEA = 0.10; CFI = 0.76 and NNFI = 0.70).

For the exploratory factor analysis, Bartlett’s sphericity test rejected the null hypothesis that the data correlation matrix was an identity matrix (p < 0.001), while Kaiser-Meyer-Olkin (KMO) was 0.643. These results show good fit of the data matrix to the factor analysis, indicating that the analysis of principal components could be performed. 

The analysis of the principal components using a scree plot resulted in three factors that explained 48.6% of the total variance, while each presented eigenvalues greater than 1 (2.20, 1.32 and 3.80) and explained 14.48%, 8.74% and 25.28% of the scale’s variance, respectively. Table 3 presents the results of the exploratory factor analysis, considering the number of factors identified in the scree plot test. The presentation of factor loads was made according to the order of the items in the factor.

**Table 3:** Analyses of the exploratory factor loads, communality (*h* *^2^* ), eigenvalues and variances for the total and each factor of the adapted version of the Appraisal of Self Care Agency Scale-Revised (n = 150). Uberaba, MG, Brazil, 2012

Scale’s items	Factor loads			*h^2^*
	1	2	3	
Item 1	0.76	0.06	-0.05	0.58
Item 2	0.55	-0.45	0.07	0.50
Item 3	0.75	0.02	0.10	0.57
Item 5	0.64	-0.04	0.36	0.54
Item 6	0.12	-0.017	0.49	0.28
Item 10	0.41	-0.004	0.40	0.33
Item 7	0.39	0.30	0.63	0.64
Item 8	0.71	0.12	0.07	0.52
Item 9	-0.03	0.64	0.14	0.44
Item 12	0.20	0.27	0.54	0.41
Item 13	-0.17	-0.64	0.11	0.45
Item 4	-0.05	0.63	0.17	0.45
Item 11	0.02	0.06	0.68	0.46
Item 14	-0.01	0.07	0.83	0.70
Item 15	0.01	0.35	0.57	0.45
Eigenvalues	2.20	1.32	3.80	
Variance explained for each factor	14.48%	8.74%	25.28%	
Total variance explained	48.6%			

Extraction method: Main components analysis; Rotation method: Varimax with Kaiser normalization; Rotation A converged in five interactions

According to the exploratory factor analysis, the reallocation of items in the factors were as follows: factor 1 “items ASAS-R 1,2,3,5,8,10”; factor 2 “items ASAS-R 4,9,13” and factor 3 “items ASAS-R 6,7,11,12,14,15”. Factor 2 “Developing power for self-care” presented the least variance for each factor (8.74%) and the smallest number of items allocated in the factor theoretically proposed (Table 3).

The factor loads were greater than 0.40 for all the items. Items ASAS-R 2 and 10 should be disregarded because they obtained a high load in more than one factor, though they were allocated to the factor with the highest load. Item ASAS-R 14 presented the greatest communality; that is, 70.0% of its variance was explained by the factors (Table 3).

In regard to the confirmatory factor analysis of the items in the factors obtained in the exploratory factor analysis, the results concerning the alpha of the items in the three factors and item-to-factor correlation coefficients were slightly better when compared to those obtained during the confirmatory factor analysis of the original structure. 

The values concerning the analysis of internal consistency were: factor 1 total alpha = 0.75; factor 2 total alpha = 0.47 and factor 3 total alpha = 0.75. Most correlations among the items of each of the three factors presented moderate to strong magnitude (r = 0.37 to r = 0.64), except for the items in factor 2 “Developing power” (ASAS-R 4, 9 and 13), which presented values below 0.30. Note that the alpha value and the item-to-factor correlation coefficients of factor 2 “Developing power” remained unsatisfactory.

## Discussion

The first version of the Appraisal of Self-Care Agency Scale (ASAS) was developed by a group of American and Dutch researchers, who belonged to the Nursing Development Conference Group (NDCG), to measure the central concept of Orem’s Self-Care Deficit Theory in1986^10^.

Even though the ASAS is based on the ten power components, it does not mention dimensions. Measurement is taken in a global and nonspecific way and can be applied and compared to different age groups under various health conditions^10^. Since then, studies have been conducted to verify the factor structure and internal consistency of the scale’s items in different countries, to meet criteria concerning construct validity^4^ ^,^ ^11^ ^,^ ^18^ ^-^ ^21^.

One study conducted with a sample of Americans with *diabetes mellitus* taking insulin verified that weak correlations found for some items suggested that the scale could have more than one dimension^4^. The authors continued the studies and decided to verify the exploratory and confirmatory factor structure of the ASAS with 24 items for a sample of 389 American individuals from the general population^11^.

The aforementioned study reports a new structure that obtained excellent goodness-of-fit index after excluding nine items and describing and listing three factors^11^. Comparison of the confirmatory factor analyses among the versions: ASAS 24 items with a single factor; ASAS 24 items with three factors; and ASAS 15 items with three factors, revealed that the last version presented the best goodness of fit, as well as the best construct validity, strongest factor loads, a high variance explained for all the items, and high reliability, in addition to showing strong linear correlation with the original (r = 0.98; p < 0.001)^11^.

Therefore, based on psychometric analysis of validation and reliability, a new version with 15 items called Appraisal of Self Care Agency-Revised (ASAS-R) was established. One of the conclusions reached by the aforementioned study was that there was a need to conduct further studies seeking to perform psychometric assessments among people with chronic diseases, especially *diabetes mellitus* ^11^.

In this sense, based on the revised version, ASAS-R, applied to a sample of Brazilian individuals with *diabetes mellitus* ^9^, this study sought to continue the validation process, analyzing correlations, internal consistency, and the results of the hypothesized model’s overall fit, so that these results could be compared with those from the original version^11^.

The correlations among the items of each of the three factors in this study presented from moderate to strong magnitude, with the exception of the items from factor 2 “Developing power for self-care”. The correlations reported by the study conducted with the original version were also of moderate to strong magnitude, though in this case, among the items of the three factors (r = 0.41 to r = 0.60, de r = 0.34 to r = 0.61 and from r = 0.40 to r = 0.57, respectively)^11^.

The results concerning the analysis of the total internal consistency of the items in the adapted version of ASAS-R (Cronbach’s alpha = 0.74) and factors 1 “Having power for self-care” and 3 “Lacking power for self-care” (Cronbach’s alphas of 0.69), were considered satisfactory, except for factor 2 “Developing power for self-care” (Cronbach’s alpha = 0.38).

The results concerning the internal consistency of the ASAS-R original version were: total alpha = 0.89 and the alphas among factors were 0.86, 0.83 and 0.79, respectively^4^, the highest total alpha, compared to studies that used the ASAS version with 24 items (total from 0.59 to 0.80)^11^.

In regard to the confirmatory factor analysis of the adapted scale, despite the weak correlations and unsatisfactory alpha value contained in factor 2, “Developing power for self-care”, the proposed theoretical model was not rejected by the χ^2^ test or the other three adequacy tests (χ^2^/g.l=2.97 GFI=0.85; RMR = 0.07 RMSEA = 0.09). The factor loads presented values greater than 0.40, except in items ASAS-R 9 and ASAS-R 13.

The study conducted with the original version^11^ ^)^ yielded greater model goodness-of-fit values in all the adequacy tests (χ^2^/g.l = 1.97; GFI = 0.94; AGFI = 0.92; CFI = 0.96; TLI = 0.95; RMSEA = 0.05; RMR = 0.05), with factor loads from 0.58 to 0.73 and explained variance from 0.34 to 0.55. Note that all items of the original version presented high factor loads, as well as satisfactory results of item-to-factor correlation, including item ASAS-R 13.

Expecting to identify the items that could be affecting reliability and the quality of the model’s fit, Wald’s and Lagrange’s multiplier’s tests were performed together with exploratory factor analysis. Even after reallocating or removing some items, however, the estimation of the factor loads and differences in the χ^2^ statistics and goodness-of-fit measures obtained in a new confirmatory analysis were not significant enough to suggest any adjustment in the specified factorial model.

In the exploratory factor analysis, the scree plot test suggested three factors, the same number presented in the original version^11^, but factor 2 still presented weaker correlations and low internal consistency, as well as a low variance was explained for each factor (8.74%).

Given the preceding discussion, it is desirable for this scale to be applied in samples from the general population to advance its development and present more evidence to strengthen analysis of the internal consistency and dimensionality of the factor structure. Additionally, we do not know the extent to which the sample’s homogeneity, in terms of sociodemographic, cultural, clinical characteristics, or in terms of accessibility to public services, contributed to the reliability results or the goodness of fit of this scale. 

## Conclusion

Analyses of product-moment correlation and reliability of the factor structure of the adapted ASAS-R were satisfactory, except for factor 2 “Developing power for self-care”. The construct validity, assessed through confirmatory factor analysis, presented satisfactory results in three goodness-of-fit indexes (GFI, RMR and RMSEA), such that it is acceptable in the proposed theoretical model. The factor loads were greater than 0.40, except for two items.

Additional statistical tests were used to improve the performance of the factor structure but the estimated values of the factor loads and goodness-of-fit measures suggested that the results of the model proposed by the authors of the original version should be maintained.

Therefore, the conclusion is that the initial factor structure of the adapted scale presented satisfactory results concerning reliability and validity but further studies are necessary. This study is expected to contribute to research addressing the concept of self-care agency and the development of ASAS-R and to favor the monitoring of individuals with DM within the care model of the public Brazilian Health System.
